# Evaluation of aloe extracts as a photosensitizer in antimicrobial photodynamic therapy against *Porphyromonas gingivalis*: An *in vitro* study

**DOI:** 10.6026/973206300221669

**Published:** 2026-03-31

**Authors:** Allen Naorem, Vishakha Patil, Vidya Dodwad, Priyanka Zerwal

**Affiliations:** 1 Department of Periodontology, Bharati Vidyapeeth (deemed to be university) Dental College and Hospital, Pune, Maharashtra, India

**Keywords:** Antimicrobial photodynamic therapy (aPDT), aloe extract, *Porphyromonas gingivalis*, natural photosensitizer, periodontitis

## Abstract

Antimicrobial photodynamic therapy (aPDT) is a promising non-antibiotic approach for periodontal disinfection, but the efficacy of natural photosensitizers like aloe extract remains underexplored. Therefore, it is of interest to evaluate the antimicrobial efficacy of aloe extract against *Porphyromonas gingivalis* under varying concentrations and irradiation durations. Results showed significant reductions in bacterial growth, with complete inhibition at 90 seconds of irradiation. The 100 µM/ml concentration demonstrated the highest efficacy at 60 seconds. It enhances understanding by demonstrating the antimicrobial efficacy of aloe extract against *Porphyromonas gingivalis* under specific conditions, which had not been thoroughly explored before.

## Background:

Periodontitis is a chronic inflammatory disease that leads to the progressive destruction of the periodontal supporting tissues, which includes the gums, bone and ligaments surrounding the teeth [1]. The disease is primarily initiated by the accumulation of pathogenic biofilms in the oral cavity, particularly around the teeth and gums. Among the many microorganisms associated with periodontitis, *Porphyromonas gingivalis*, a Gram-negative anaerobic bacterium, plays a central role as a keystone pathogen [2]. This bacterium is notorious for its ability to manipulate host immune responses, creating a pro-inflammatory environment that facilitates disease progression. It also promotes microbial dysbiosis, an imbalance in the composition of the oral microbiota, which further exacerbates the disease [3]. Conventional periodontal therapy typically involves mechanical debridement of the periodontal pockets, aimed at removing the accumulated plaque and tartar. This is often supplemented with antimicrobial agents to target any remaining pathogenic bacteria [4]. However, the use of antibiotics in periodontal therapy has raised significant concerns in recent years. The overuse and misuse of antibiotics have contributed to the global rise of antibiotic resistance, which not only undermines the effectiveness of standard therapies but also poses significant health risks, including the development of multidrug-resistant pathogens [5]. Moreover, the use of antibiotics in periodontal treatment can have adverse effects on the oral microbiome, potentially leading to further complications. This has sparked the need for alternative, non-antibiotic treatment modalities that can effectively manage periodontitis without the risk of resistance or other side effects [6]. One promising alternative is antimicrobial photodynamic therapy (aPDT), a technique that employs light-activated compounds known as photosensitizers to selectively target and eliminate microorganisms. In aPDT, a photosensitizer is activated by light of a specific wavelength, leading to the production of reactive oxygen species (ROS) when in the presence of oxygen [7]. These ROS induce cytotoxic effects that damage the microbial cells, leading to their destruction. aPDT has garnered attention as a potential adjunctive therapy for periodontitis due to its ability to target bacteria directly while minimizing damage to surrounding tissues [8]. The interest in natural photosensitizers, such as Aloe vera, has been growing due to their favorable safety profile. Aloe vera, a widely used medicinal plant, contains anthraquinone derivatives like aloe emodin, which have demonstrated both photoactive and antimicrobial properties [9]. These compounds can absorb light and convert it into energy, making them suitable for use as photosensitizers in aPDT. Given the antimicrobial activity of aloe vera and its biocompatibility, it presents a promising candidate for periodontal disinfection, particularly against pathogens like *P. gingivalis* that are resistant to conventional therapies [10]. Therefore, it is of interest to report to determine the effectiveness of aloe extract as a natural and biocompatible photosensitizer in aPDT, specifically targeting *Porphyromonas gingivalis* and to evaluate its potential as a safe and efficient adjunctive treatment for periodontitis.

## Methodology:

This *in vitro* experimental study was conducted at the Maratha Mandal Research Center, Belagavi, following approval from the Institutional Ethics Committee of Bharati Vidyapeeth (Deemed to be University) Dental College and Hospital, Pune. Aloe extract powder was dissolved in dimethyl sulfoxide to obtain a master solution of 100 mM. Working solutions of 15 µM/ml, 50 µM/ml and 100 µM/ml were prepared using phosphate buffered saline. *Porphyromonas gingivalis* (ATCC 33277) was cultured anaerobically and adjusted to 0.5 McFarland standards. The inoculum was dispensed into 96 well microtiter plates. Following dark incubation for 30 minutes, samples were irradiated using a blue LED unit (400-500 nm) for 0, 60, or 90 seconds, maintaining a standardized distance of 5 mm. Post irradiation, samples were plated on blood agar and incubated anaerobically for 48 hours. CFU counts were recorded. Data were analysed using SPSS version 25. Normality was assessed using Kolmogorov-Smirnov and Shapiro-Wilk tests. Intragroup comparisons were performed using paired t tests and intergroup comparisons were analysed using one way ANOVA with Tukey's post hoc test. A p value < 0.05 was considered statistically significant.

## Results:

The antimicrobial efficacy of Aloe extract as a photosensitizer in antimicrobial photodynamic therapy (aPDT) was evaluated by measuring the reduction in *Porphyromonas gingivalis* colony-forming units (CFUs) after varying irradiation durations (0, 60 and 90 seconds). A statistically significant reduction in CFU counts was observed for all aloe extract concentrations (15 µM/ml, 50 µM/ml and 100 µM/ml) as the irradiation time increased (p < 0.05). At 90 seconds of blue LED light irradiation, complete inhibition of *P. gingivalis* growth was achieved for all concentrations. At 60 seconds, the 100 µM/ml concentration exhibited the highest antimicrobial efficacy, showing a significantly greater reduction in CFUs compared to the 15 µM/ml and 50 µM/ml concentrations (p < 0.05). The results demonstrated that both aloe extract concentration and irradiation duration significantly influence the antimicrobial effect of aPDT on *P. gingivalis*. Table 1 (see PDF) shows intragroup comparison of CFU counts of *P. gingivalis* at different irradiation durations (0, 60 and 90 seconds) for Aloe extract concentrations (15, 50 and 100 µM/ml). Table 2 (see PDF) shows intergroup comparison of antimicrobial efficacy (CFU/ml) among Aloe extract concentrations at 60 seconds of blue LED irradiation.

## Discussion:

The findings of the present study demonstrate that aloe extract can effectively function as a photosensitizer in aPDT against *P. gingivalis*. The enhanced antimicrobial effect observed with increased irradiation time is consistent with the photodynamic mechanism involving reactive oxygen species generation. Natural photosensitizers such as aloe extract offer advantages of low dark toxicity and additional biological benefits, supporting their potential role in periodontal therapy. However, extrapolation of these *in vitro* results to clinical settings warrants further *in vivo* and clinical studies. Moslemi *et al.* (2018) [11] investigated aPDT using Radachlorin and Toluidine Blue O (TBO) against *P. gingivalis* and found that combining a photosensitizer with light significantly reduced bacterial viability compared with photosensitizer or light alone, with laser based aPDT showing superior efficacy. Similarly, your results demonstrated that increasing irradiation time enhanced antimicrobial effect, highlighting that photosensitizer + light yields greater pathogen reduction than controls (Radachlorin + laser > TBO + LED). Shahmoradi *et al.* (2023) [12] showed that dendrosomal curcumin as a natural photosensitizer with blue laser aPDT significantly reduced *P. gingivalis* growth *in vitro*, with complete inhibition at 90 s similar to your findings where 90 s irradiation with Aloe extract achieved total bacterial inhibition, supporting time dependent photokilling and natural compounds efficacy. Pan *et al.* (2020) [13] evaluated curcumin based aPDT against *P. gingivalis* and observed a significant decrease in bacterial counts with blue LED irradiation plus curcumin compared to untreated or light alone, aligning with your data on reduced CFU with photosensitizer + light and further validating blue light activation of natural photosensitizers yields antimicrobial effects. Oruba *et al.* (2021) [14] reported that TBO mediated aPDT dramatically reduced viability of intracellular *P. gingivalis* with sufficient light exposure, emphasizing that effective light activated photosensitization can significantly kill periopathogens - a concept confirmed by your results showing enhanced killing with increasing irradiation durations. Yoshida *et al.* (2021) [15] demonstrated that *P. gingivalis* was more susceptible to blue LED activated Rose Bengal (RB) mediated a PDT than to methylene blue and that bactericidal effects increased in a time dependent manner, as singlet oxygen production rose a pattern consistent with your findings of significant CFU reductions with longer irradiation and higher concentration.

## Conclusion:

Aloe extract-mediated antimicrobial photodynamic therapy using blue LED light effectively reduces *Porphyromonas gingivalis* growth *in vitro*. Both higher concentrations and longer irradiation durations significantly improve antimicrobial outcomes. Thus, we show that aloe extract could serve as a promising natural adjunct in periodontal therapy.
